# Supplementary material for: Modeling glioblastoma heterogeneity as a dynamic network of cell states
Source: Mol Syst Biol. 2021 Sep 16;17(9):e10105. doi: 10.15252/msb.202010105 (PMC8444284; doi:10.15252/msb.202010105)
Supplement: Supplementary file 6 — Source Data for Figure 5 [file MSB-17-e10105-s004.zip › Figure5A_sourcedata/GSEA_3071/hallmarks_stateIV.GseaPreranked.1623742551702/HALLMARK_ALLOGRAFT_REJECTION.html]

Details for gene set HALLMARK\_ALLOGRAFT\_REJECTION[GSEA]

|  || Dataset | state53071 |
| Phenotype | NoPhenotypeAvailable |
| Upregulated in class | na\_neg |
| GeneSet | HALLMARK\_ALLOGRAFT\_REJECTION |
| Enrichment Score (ES) | -0.41997838 |
| Normalized Enrichment Score (NES) | -1.977417 |
| Nominal p-value | 0.0018484289 |
| FDR q-value | 0.0018855104 |
| FWER p-Value | 0.021 |
Table: GSEA Results Summary

  

Fig 1: Enrichment plot: HALLMARK\_ALLOGRAFT\_REJECTION      
 Profile of the Running ES Score & Positions of GeneSet Members on the Rank Ordered List

  

| PROBE | GENE SYMBOL | GENE\_TITLE | RANK IN GENE LIST | RANK METRIC SCORE | RUNNING ES | CORE ENRICHMENT || 1 | TGFB2 |  |  | 108 | 1.511 | 0.0199 | No |
| 2 | HDAC9 |  |  | 510 | 0.810 | -0.0350 | No |
| 3 | ST8SIA4 |  |  | 567 | 0.751 | -0.0256 | No |
| 4 | IL7 |  |  | 1038 | 0.509 | -0.1018 | No |
| 5 | NCK1 |  |  | 1070 | 0.502 | -0.0943 | No |
| 6 | CSF1 |  |  | 1189 | 0.469 | -0.1042 | No |
| 7 | GALNT1 |  |  | 1314 | 0.437 | -0.1162 | No |
| 8 | BCL3 |  |  | 2125 | 0.280 | -0.2634 | No |
| 9 | AKT1 |  |  | 2211 | 0.267 | -0.2725 | No |
| 10 | APBB1 |  |  | 2278 | 0.258 | -0.2782 | No |
| 11 | ETS1 |  |  | 2305 | 0.254 | -0.2763 | No |
| 12 | CSK |  |  | 2331 | -0.250 | -0.2744 | No |
| 13 | B2M |  |  | 2349 | -0.252 | -0.2709 | No |
| 14 | RPL39 |  |  | 2419 | -0.260 | -0.2771 | No |
| 15 | DYRK3 |  |  | 2475 | -0.267 | -0.2805 | No |
| 16 | TRAF2 |  |  | 2680 | -0.293 | -0.3116 | No |
| 17 | BCL10 |  |  | 2868 | -0.315 | -0.3389 | No |
| 18 | EIF3A |  |  | 2885 | -0.317 | -0.3335 | No |
| 19 | JAK2 |  |  | 2912 | -0.320 | -0.3298 | No |
| 20 | ELF4 |  |  | 3036 | -0.337 | -0.3443 | No |
| 21 | MTIF2 |  |  | 3041 | -0.338 | -0.3360 | No |
| 22 | CDKN2A |  |  | 3146 | -0.353 | -0.3464 | No |
| 23 | IFNGR1 |  |  | 3532 | -0.415 | -0.4089 | Yes |
| 24 | HIF1A |  |  | 3542 | -0.418 | -0.3994 | Yes |
| 25 | NME1 |  |  | 3548 | -0.419 | -0.3891 | Yes |
| 26 | GLMN |  |  | 3613 | -0.431 | -0.3898 | Yes |
| 27 | PSMB10 |  |  | 3629 | -0.433 | -0.3811 | Yes |
| 28 | TLR3 |  |  | 3642 | -0.435 | -0.3717 | Yes |
| 29 | ITGB2 |  |  | 3649 | -0.435 | -0.3612 | Yes |
| 30 | CTSS |  |  | 3693 | -0.443 | -0.3576 | Yes |
| 31 | GZMB |  |  | 3735 | -0.451 | -0.3533 | Yes |
| 32 | ABCE1 |  |  | 3867 | -0.480 | -0.3655 | Yes |
| 33 | CCND3 |  |  | 3901 | -0.487 | -0.3587 | Yes |
| 34 | RIPK2 |  |  | 4107 | -0.542 | -0.3834 | Yes |
| 35 | IL15 |  |  | 4186 | -0.564 | -0.3832 | Yes |
| 36 | BCAT1 |  |  | 4272 | -0.590 | -0.3836 | Yes |
| 37 | DEGS1 |  |  | 4281 | -0.592 | -0.3693 | Yes |
| 38 | TAP2 |  |  | 4315 | -0.602 | -0.3595 | Yes |
| 39 | F2R |  |  | 4396 | -0.634 | -0.3578 | Yes |
| 40 | EIF3J |  |  | 4426 | -0.643 | -0.3461 | Yes |
| 41 | EIF3D |  |  | 4523 | -0.692 | -0.3459 | Yes |
| 42 | THY1 |  |  | 4587 | -0.729 | -0.3384 | Yes |
| 43 | EIF5A |  |  | 4738 | -0.821 | -0.3451 | Yes |
| 44 | TGFB1 |  |  | 4781 | -0.846 | -0.3304 | Yes |
| 45 | CAPG |  |  | 4812 | -0.877 | -0.3127 | Yes |
| 46 | LIF |  |  | 4841 | -0.907 | -0.2937 | Yes |
| 47 | BRCA1 |  |  | 4938 | -1.002 | -0.2852 | Yes |
| 48 | FLNA |  |  | 4975 | -1.051 | -0.2640 | Yes |
| 49 | NPM1 |  |  | 5048 | -1.184 | -0.2460 | Yes |
| 50 | TIMP1 |  |  | 5086 | -1.257 | -0.2194 | Yes |
| 51 | SOCS1 |  |  | 5138 | -1.391 | -0.1919 | Yes |
| 52 | IL11 |  |  | 5219 | -1.763 | -0.1600 | Yes |
| 53 | IL18 |  |  | 5224 | -1.814 | -0.1122 | Yes |
| 54 | EGFR |  |  | 5233 | -1.904 | -0.0627 | Yes |
| 55 | INHBA |  |  | 5280 | -2.740 | 0.0019 | Yes |
Table: GSEA details [plain text format]

  

Fig 2: HALLMARK\_ALLOGRAFT\_REJECTION: Random ES distribution      
 Gene set null distribution of ES for **HALLMARK\_ALLOGRAFT\_REJECTION**
